# Risk factors for post-weaning diarrhoea on piglet producing farms in Finland

**DOI:** 10.1186/1751-0147-50-21

**Published:** 2008-06-18

**Authors:** Taina M Laine, Tapani Lyytikäinen, Maija Yliaho, Marjukka Anttila

**Affiliations:** 1 Finnish Food Safety Authority Evira, Production Animal Health Unit, Mustialankatu 3, FI-00790 Helsinki, Finland; 2 Finnish Food Safety Authority Evira, Risk Assessment Unit, Mustialankatu 3, FI-00790 Helsinki, Finland; 3 Rural Advisory Centre of Southern Ostrobothnia, Huhtalantie 2, FI-60220 Seinäjoki, Finland; 4 Finnish Food Safety Authority Evira, Pathology Unit, Mustialankatu 3, FI-00790 Helsinki, Finland

## Abstract

**Background:**

Post-weaning diarrhoea (PWD) is a significant gastrointestinal disease in pigs. It is considered a multifactorial disease associated with proliferation of enterotoxigenic *Escherichia coli *in the intestinal tract of affected pigs. The aim of this study was to analyse risk factors related to the occurrence of PWD on Finnish piglet producing farms.

**Methods:**

The data of a follow-up study of 73 conventional piglet producing farms was used in the case-control study. The selection of the 41 PWD case and 28 control farms was based on the use of antimicrobials for treating diarrhoea in weaned pigs and the answers related to the occurrence of diarrhoea after weaning in the questionnaire. Four intermediate farms were excluded from the statistical analysis.

Altogether 39 factors related to herd characteristics, weaner pig management and pig health were studied. The median number of sows was 59.0 (IQR = 44.0; 74.5) and 52.5 (IQR = 36.8; 61.5) on the case and the control farms, respectively.

The significances of the univariable associations between the explanatory variables and the outcome variable were tested, and in the multivariate analysis quasibinomial generalized linear models were applied.

**Results:**

An increased risk of PWD was associated with the regimen of twice a day feeding and feed restriction after weaning (*P *= 0.02; compared to feeding three or more meals a day or the use of *ad libitum *feeding) and with a higher number of sows on the farm (*P *= 0.02; risk increasing with increasing number of sows). Automatic temperature control was associated with a decreased risk of PWD (*P *= 0.03; compared to manual temperature control).

**Conclusion:**

Twice a day feeding of newly-weaned pigs should be avoided if the amount of feed given is restricted. Variation in ambient temperature should be minimized in housing of newly-weaned pigs and this can be achieved by using automatic temperature control. With increasing number of sows in the herds the risk of PWD increases and more attention should be paid to prevention of post-weaning diarrhoea.

## Background

Antimicrobial resistance in pathogenic bacteria is a global threat and therefore increasing attention is being paid to the prudent use of antibiotics in food-producing animals [1]. Gastrointestinal diseases of growing pigs are economically important for pig production worldwide [2] and enteric bacterial infections are often treated with antimicrobials. Detailed data registered in 2004 by the VetStat programme on antimicrobial use in Denmark showed that prescriptions for weaner pigs accounted for more than one third of the total antimicrobial consumption in pigs and that gastrointestinal diseases were the most common indications for prescriptions in this age group [3].

PWD is considered to be a multifactorial disease [4,5].

Recently weaned pigs are variously predisposed to enteric disorders. Newly weaned pigs are stressed by nutritional, psychological, environmental and physiological factors [4]. At weaning the feed is changed from milk to a weaner diet, piglets are separated from their sow and often moved from the farrowing pen and mixed with unfamiliar pigs. Weaned pigs also lose passive intestinal immunity provided by antibodies in sow's milk [6]. After weaning there are alterations in the structure [7] and function [8,9] of the piglet small intestine, changes in intestinal *E. coli *flora of piglets [10,11] and impairment of immune functions in early-weaned piglets [12,13].

During the first two weeks after weaning, pathogenic *Escherichia coli *plays a significant role in the etiology of PWD [5,14,15], although infection with pathogenic *E. coli *does not unequivocally lead to the development of diarrhoea in weaned pigs [5,16-18].

Pens contaminated with pathogenic *E. coli *strains are likely sources of infection for weaned pigs, but the infection can also be acquired before weaning [19].

On farms outbreaks of PWD can occur suddenly and during outbreaks the morbidity may be over 50% among weaned piglets [14,15]. Severely affected pigs can die acutely [14,20]. In surviving pigs diarrhoea can be transient [5] or it can last for up to four days [20]. The case fatality rate seldom exceeds 10% in uncomplicated cases [21].

PWD associated with enterotoxigenic *E. coli *typically affects pigs during the immediate post-weaning period. In contrast to PWD, the enteric diseases caused by *Lawsonia intracellularis *and *Brachyspira *– bacteria also affect growers and fattening pigs [22,23].

Several factors have been reported to influence the occurrence of diarrhoea in weaned pigs. The susceptibility for diarrhoea after weaning has been associated with management related factors such as low feed intake during the first week after weaning [24], excessive feed intake [16], low number of meals [25], the hygiene and management level [24], low weaning weight and age [26,27], moderate cold stress [17], draught [28], texture of feed [29], number of feeder spaces per pen [29] and vaccinating gestating sows against PRRS (porcine reproductive and respiratory syndrome) [29]. Cleaning pens between batches of weaned pigs has been associated with a decreased risk of PWD [30].

In Finland the use of antimicrobial growth promoters (AGPs) was voluntarily stopped soon after the use of olaquindox and carbadox was banned in the European Union in 1999. Until the ban these two substances had been added to commercial weaner feeds also in Finland.

In piglet production without AGPs knowledge of the relationship between management related factors and occurrence of diarrhoea in weaned pigs is important. Management related measures can be used in prevention of PWD [4] and finding specific solutions for each farm has been emphasized [31].

This study was done to provide information on risk factors related to the occurrence of PWD on Finnish piglet producing farms after the AGP withdrawal.

## Methods

### Study sample

The farms were recruited among herds that used a production-data recording system run by the Association of the Rural Advisory Centres. A total of 260 piglet producing farms were supervised by these centers in 1999. Animal production advisors of the Rural Advisory Centres provided the farms located in four different provinces. Random sampling was not possible, because all the farms that were willing to participate were needed. In the beginning the herds had either few or moderate problems with post-weaning diarrhoea based on the evaluation of the farmer. It was not possible to select the case and the control farms directly because before the study began, there was no comprehensive knowledge about the occurrence of post-weaning diarrhoea on Finnish piglet-producing farms.

Our case-control study is based on the material of the follow-up study of 73 conventional piglet producing farms in 1999–2000 in which data related both to the occurrence of diarrhoea and to antimicrobial treatments for diarrhoea after weaning were collected. The total follow-up period was either 16 months (33 farms) or 12 months (40 farms) and it was divided into consecutive 4-month periods [32].

The follow-up data were used to assign the case and the control farms related to the occurrence of PWD. A total of 41 farms were defined as PWD cases and 28 farms as control farms. Four farms were classified being intermediate relative to occurrence of PWD and these farms were excluded from the statistical analysis.

Two types of herds were included in the case-control study: 41 farrowing herds and 28 farrow-to-finish herds. A farrowing herd only raises piglets to the mean body weight of about 25 kg and after that feeder pigs are delivered to specialised finishing herds. Farrow-to-finish herds raise their own finishing pigs.

Concerning the health status of the farms, Finland is considered free of classical swine fever, swine vesicular disease, Aujeszky's disease and transmissible gastroenteritis (TGE) and porcine reproductive and respiratory syndrome (PRRS) has not been reported in Finland [33].

### Data collection

The follow-up data were used both for the definition of the PWD cases and the control farms and for obtaining data on explanatory variables.

The data on the occurrence of PWD were collected with help of the questionnaire and from records the farmers had kept on cases of diarrhoea after weaning and antimicrobial treatments related to them. The questionnaire included separate closed questions both about the occurrence of diarrhoea in pigs within 14 days after weaning and diarrhoea over 14 days after weaning. The farmers were asked to answer these questions based on their experiences during a few previous months. The given alternatives were: no cases, seldom/occasionally, periodically and regularly. During the follow-up in a 4-month period the use of antimicrobials for treatment of diarrhoea after weaning was calculated as the number of piglets treated divided by the number of piglets weaned.

Data on explanatory variables were obtained from the production-data recording system (number of sows, the average age at weaning, piglets weaned/sow/year) and from the questionnaire (farm management related to weaning of piglets; the environment of weaned pigs; health status related to *Mycoplasma hyopneumoniae *and *Sarcoptes scabiei*; sow vaccination, observations concerning health of weaned pigs). The questionnaire consisted of 53 questions, and mainly closed questions were used. The data of one questionnaire per farm was used in the analysis. During the follow-up study animal production advisors visited the farms three times and every time a similar questionnaire was filled. These advisors of seven different Rural Advisory Centres and one slaughter-house co-operative also normally visited the farms and collected data for production monitoring run by the Association of the Rural Advisory Centres. A total of 33, 27, 6 and 3 questionnaires were responded in March-May, June-August, September-November and December-February, respectively. For the case herds the questionnaire was used that was responded during a time when most severe problems with PWD were experienced according to the follow-up data. The classification of the farms was done retrospectively and at the time of the farm visits the advisors were not aware of the herd status (as case or control). Eight advisors accounted for 61 questionnaires filled on both PWD case and control farms and four advisors accounted each a questionnaire on either 1–3 case or control farms.

### Definition of outcome variable

The unit of observation was the farm. Based on the retrospective follow-up data the farms were divided into control farms that experienced few problems related to post-weaning diarrhoea and PWD case farms that had moderate to severe problems related to post-weaning diarrhoea. The outcome variable was thus dichotomous.

On the 28 control farms the questionnaire was responded in a 4-month follow-up period during which these farms had treated less or no more than 5% of weaned piglets with antimicrobials for diarrhoea. In addition, these farms gave answers 'no cases' or 'seldom/occasionally' in the questionnaire related to the occurrence of diarrhoea after weaning.

The definition of the PWD case farms was based on antimicrobial treatments for diarrhoea and experienced problems related to PWD. The two alternative criteria were: 1) The questionnaire was responded in a 4-month follow-up period (or just after it) during which the farm treated at least 10% or more of the weaned piglets with antimicrobials for diarrhoea after weaning (34 cases) or 2) In the questionnaire the farm responded 'periodically' or 'regularly' to the questions related to the occurrence of diarrhoea after weaning (7 cases).

Four farms with intermediate data on antimicrobial use for diarrhoea after weaning (6–9%) in the 4-month periods of the questionnaires and with answers 'seldom/occasionally' related to the occurrence of diarrhoea after weaning were excluded from the statistical analysis.

### Definition of the explanatory variables

Tables 1 and 2 list the variables under study. Due to the evident synergism and/or collinearity three combined variables were generated. Feeding regimen was divided into two categories by combining restricted feeding after weaning and providing feed only twice a day to newly-weaned pigs to be one category (F-R2M) and other feeding regimens (*ad libitum *feeding, at least three meals per day with or without feed restriction) to be the other (F-OTHER). The two other combined explanatory variables generated as indices are shown in Table 3.

**Table 1:** Continuous explanatory variables on 41 post-weaning diarrhoea case farms and 28 control farms. IQR, Interquartile range.

	**PWD cases**		**Controls**		***P***
			
	**Median**	**IQR**	**Median**	**IQR**	
Age at weaning	33.0	30.5; 36.0	34.5	30.0; 36.8	0.47
Environmental temperature for the weaners	21	20; 23	22	20;23	0.69
Temperature in the lying area for the weaners	24	22; 26	24	22;27	0.99
Number of sows	59.0	44.0; 74.5	52.5	36.8; 61.5	0.07
Piglets weaned/sow/year	18.9	17.4; 20.9	18.5	17.5; 20.5	0.98

**Table 2:** Non-continuous explanatory variables on 41 post-weaning diarrhoea case farms and 28 control farms.

**Explanatory variable**	**Categories**	**Number (%) of herds**		***P-*value**
		
		**Cases**	**Controls**	
***Herd***				
Type of herd				0.75
	Farrowing herd	25 (61.0)	16 (57.1)	
	Farrow-to-finish herd	16 (39.0)	12 (42.9)	
The number of sows increased by 50% or more from 1998 to 2000				0.58^e^
	Yes	16 (39.0)	12 (42.9)	
	No	23 (56.1)	13 (46.4)	
	No data on 1998	2 (4.9)	3 (10.7)	
***Pen and environment***				
Floor type in the farrowing pens				NE^c^
	Solid	28 (68.3)	21 (75.0)	
	Partly slatted	9 (22.0)	7 (25.0)	
	Fully slatted	4 (9.7)	0	
Floor type in the nursery pens				0.23^c^
	Solid	14 (34.2)	11 (39.3)	
	Partly slatted	20 (48.8)	16 (57.1)	
	Fully slatted	6 (14.6)	1 (3.6)	
	Deep bedding	1 (2.4)	0	
Use of bedding for newly-weaned pigs				0.06
	Yes	30 (73.2)	26 (92.9)	
	No	11 (26.8)	2 (7.1)	
Heating in the accomodation for weaners				1.00
	Yes	39 (95.1)	27 (96.4)	
	No	2 (4.9)	1 (3.6)	
Temperature control				0.12
	Automatic	17 (41.5)	17 (60.7)	
	Manual	24 (58.5)	11 (39.3)	
Heated lying area for weaners				0.40
	Yes	37 (90.2)	27 (96.4)	
	No	4 (9.8)	1 (3.6)	
***Animal husbandry and hygiene***				
Piglets moved from the farrowing pen within 7 days after weaning				0.95
	Yes	12 (29.3)	8 (28.6)	
	No	29 (70.7)	20 (71.4)	
Mixing of litters after weaning				0.43
	Yes	30 (73.2)	18 (64.3)	
	No^a^	11 (26.8)	10 (35.7)	
Runts moved to groups of younger weaned piglets				0.48
	Yes	17 (41.5)	14 (50.0)	
	No	24 (58.5)	14 (50.0)	
Piglets moved within 7 days after weaning to a separate room with own airspace intended for rearing of weaners only or for rearing of weaners and growers				0.85
	Yes	8 (19.5)	6 (21.4)	
	No	33 (80.5)	22 (78.6)	
Reared in a room with own airspace for weaners and growers from 1–3 weeks after weaning up to the body weight of about 25 kg				0.60
	Yes	15 (36.6)	12 (42.9)	
	No	26 (63.4)	16 (57.1)	
HYGIND (see Table 3)				0.86
	0 – 0.5	8 (19.5)	5 (17.9)	
	1 – 1.5	8 (19.5)	7 (25.0)	
	2 – 2.5	9 (22.0)	6 (21.4)	
	3 – 3.5	11 (26.8)	5 (17.9)	
	4 – 4.5	2 (4.9)	2 (7.1)	
	5 – 6	3 (7.3)	3 (10.7)	
***Feeding and watering***				
Creep feeding				NE
	Yes	40 (97.6)	28 (100.0)	
	No	1 (2.4)	0	
Commercial feed for weaners				NE
	Yes	41 (100.0)	26 (92.9)	
	No	0	2 (7.1)	
Piglet age at change from unmixed weaner feed to other feed				0.79^f^
	6–7 weeks	28 (68.3)	19 (67.9)	
	8 weeks or older	12 (29.3)	7 (25.0)	
	No data or own feed	1 (2.4)	2 (7.1)	
A feeder as an only feed supply in the pen for newly-weaned piglets				0.18
	Yes	15 (36.6)	6 (21.4)	
	No	26 (63.4)	22 (78.6)	
Through feeding for newly-weaned piglets				0.20
	Yes	20 (48.8)	18 (64.3)	
	No	21 (51.2)	10 (35.7)	
Through feeding for older weaned pigs				0.44^e^
	Yes	25 (61.0)	20 (71.4)	
	No	15 (36.6)	8 (28.6)	
	No data	1 (2.4)		
Floor feeding				0.85
	Yes	8 (19.5)	6 (21.4)	
	No	33 (80.5)	22 (78.6)	
Feed provided for newly weaned pigs per day (times)				0.20
	Two times	21 (51.2)	10 (35.7)	
	At least 3 times or ad lib	20 (48.8)	18 (64.3)	
Special measures related to feeding after weaning				0.53^d^
	Restricted feeding	14 (34.2)	12 (42.9)	
	Restricted feeding and other^b^	18 (43.9)	8 (28.6)	
	Other^b^	6 (14.6)	1 (3.6)	
	None	3 (7.3)	7 (25.0)	
Restricted feeding and feed given twice a day for newly-weaned piglets (F-R2M)				0.17
	Yes	20 (48.8)	9 (32.1)	
	No	21 (51.2)	19 (67.9)	
Separate water supply for piglets in the farrowing pen (nipple or cup)				NE
	Yes	41 (100.0)	27 (96.4)	
	No	0	1 (3.6)	
Type of water supply in the nursery pen				NE
	Nipple	39 (95.1)	28 (100.0)	
	Cup or other	2 (4.9)	0	
***Farmer observations***				
Seasonal variation in the occurrence of PWD				0.97^e^
	Yes	15 (36.6)	10 (35.7)	
	No	26 (63.4)	17 (60.7)	
	No data		1 (3.6)	
Variation in the occurrence of PWD between pens				0.70^e^
	Yes	4 (9.8)	4 (14.3)	
	No	37 (90.2)	22 (78.6)	
	No data		2 (7.1)	
Lying behaviour of the weaners observed by the farmer				NE
	Yes	40 (97.6)	28 (100.0)	
	No	1 (2.4)	0	
***Health***				
Behavioural vices occurring often in weaned piglets	Yes	2 (4.9)	0	NE
	No	39 (95.1)	28 (100.0)	
Problems with pre-weaning diarrhoea				0.51
	Yes	7 (17.1)	3 (10.7)	
	No	34 (82.9)	25 (89.3)	
Sow vaccination against *E. coli*				0.95^e^
	Yes	24 (58.5)	16 (57.1)	
	No	17 (41.5)	11 (39.3)	
	No data		1 (3.6)	
*Mycoplasma hyopneumoniae *status				1.00
	M. hyo +	4 (9.8)	2 (7.1)	
	M. hyo -	36 (87.8)	25 (89.3)	
	Not known	1 (2.4)	1 (3.6)	
*Sarcoptes scabiei *status				1.00
	S. scab + or medication	3 (7.3)	3 (10.7)	
	S. scab -	36 (87.8)	25 (89.3)	
	Not known	2 (4.9)	0	

^a ^Moving occasionally at weaning only a few piglets from a litter to another was not categorized as mixing of litters.

^b ^Other measures related to feeding after weaning include giving electrolyte solutions, adding acid to feed, adding fibre, giving peat with iron, adding zink etc.

^c ^Fully slatted floor tested against other floor type (solid or partly slatted floor or deep bedding)

^d ^Restricted feeding regimens tested against other or no measures.

^e ^Farms with no data not included.

^f ^Farms with no data or own feed not included.

NE = non-estimable variable; one of the values is zero.

**Table 3:** Combined variables related to hygienic measures, empty time of the pens and age segregation after weaning.

**Title and definitions**	**Score**	**Scale**
**HYGIND**		**0 – 6**

**Farrowing pens**		**0 – 3**

Washed after every litter or after every two litters	1	
Disinfection after the pens have been washed	1	
Washed occasionally (e.g. few times a year)	0	
Disinfection occasionally or use of dry disinfectant	0	
Empty time ≥ 4 days	1	
Empty time 1–3 days	0.5	
Empty time < 1 day	0	

**Nursery pens**		**0 – 3**

Washed after every group or after every two groups	1	
Disinfection after the nursery pens have been washed	1	
Nursery pens washed occasionally (e.g. few times a year)	0	
Disinfection occasionally or use of dry disinfectant	0	
Deep bedding in the nursery pen	1	
Empty time ≥ 4 days	1	
Empty time 1–3 days	0.5	
Empty time < 1 day	0	

**MANIND = HYGIND + scores related to age segregation**		**0 – 8**

**Age segregation**		**0 – 2**

**First week after weaning**		

Weaners moved within 7 days after weaning to a room (with own airspace) intended for weaners only or for weaners and growers up to about 25 kg bodyweight	1	

**From 1–3 weeks after weaning up to about the body weight of 25kg**		

Weaners reared in a room (with own airspace) intended only for weaners and growers	1	

**Weaners and growers reared in a room together with older pigs (>25 kg)**	0	

### Statistical analysis

Cross tabulation and chi-square test or Fisher's exact test were used to test the significance of the univariable associations between the explanatory variables and the outcome variable. Fisher's exact test was used when expected cell frequencies were <5. The likelihood ratio chi-square test probability for a variable to be included in the multivariable analysis was set to be less than 0.20. The same probability limit was used for explanatory variables tested with Fisher's exact test. In the preliminary step in the selection of the variables the bilateral relationships between possible explanatory variables were checked to be able to lower the risk of multicollinearitity [34]. For variables evidencing a strong structural collinearity or having a statistically significant connection (e.g. totally slatted floor and use of bedding) only the variable most strongly associated with the outcome was selected.

Finally, use of bedding, feeding regimen (F-R2M), automatic temperature control in the accommodation of the weaners, a feeder as an only feed supply in the weaning pen and the average number of sows on the farm were offered to the model. The number of sows was applied as a covariate because it appeared to influence to the probability of having more problems with diarrhoea in weaned piglets. In addition, generated indices (Table 3) were included in this further analysis one at a time. In the multivariate analysis the influence of explanatory variables to the risk of experiencing problems related to post-weaning diarrhoea in piglets was studied by applying quasibinomial generalized linear models [35,36]. Calculations were done on R (2.51) [37] statistical programme, using GLM procedure, which applies iterative reweighted least squares as an estimation method [36].

All the coefficients were estimated at the average sow number of the data by centering the covariate. Significances of individual parameters were tested by multiple Wald test where the model without one of the explanatory variables was compared against the full model.

## Results

### Farms

The typical point of time for the occurrence of diarrhoea in pigs within 14 days after weaning is shown in Table 4.

**Table 4:** The typical point of time for the occurrence of diarrhoea within 14 days after weaning on the farms. The control farms had had either no cases of diarrhoea or cases of diarrhoea seldom/occasionally.

**Period for diarrhoea occurrence**	**Farms**	
	**PWD cases (%)**	**Controls (%)**

	**n = 41**	**n = 28**

No cases of diarrhoea	0 (0)	5 (17.9)
Cases of diarrhoea (also if few)		
0–4 days after weaning	5 (12.2)	1 (3.6)
5–10 days after weaning	33 (80.5)	16 (57.1)
0–10 days after weaning	2 (4.9)	0 (0)
11–14 days after weaning	1 (2.4)	2 (7.1)
Typical time period not given	0 (0)	4 (14.3)

In older weaned pigs (after the first two weeks post weaning) no cases of diarrhoea had occurred on 11 control farms (39%) and on 11 PWD case farms (27%). Three PWD case farms (7%) suffered periodically from diarrhoea over 14 days after weaning. On all the other farms cases of diarrhoea over 14 days after weaning had occurred seldom/occasionally.

No cases of oedema disease was observed in weaned pigs on 24 control farms (86%) and on 30 PWD case farms (73%) during the previous months. The rest of the farms had had cases of oedema disease seldom/occasionally.

Two case farms added zinc to weaner feed.

The median group size for pigs at about 25 kg bodyweight was 12 (IQR = 10; 20) and 10 (IQR = 10; 15) on the case farms and the control farms, respectively.

Most of the farms were free from *Mycoplasma hyopneumoniae *infection and *Sarcoptes scabiei *infestation (Table 2).

### Results of the statistical analysis

Both generated indices (HYGIND and MANIND) failed to give a statistical connection with the probability of experiencing problems related to post weaning diarrhoea (*P *= 0.86 for HYGIND; *P *= 0.40 for MANIND). Also the use of bedding appeared to have no statistically significant (*P *= 0.23) influence on the probability of experiencing problems with post-weaning diarrhoea.

The feeding regimen with feed restriction and two meals a day after weaning (F-R2M) and a higher number of sows on the farm were both associated with an increased risk of PWD. On the contrary, the presence of automatic temperature control in the accommodation of weaners seemed to be related to a decreased risk of PWD (Table 5). The 95% confidence interval of the odds ratio for the feeding regimen (F-R2M) was 1.19–19.12 and for the automatic temperature control in the accommodation of weaners it was 0.08–0.93.

**Table 5:** The parameter estimates of the final generalized linear model describing the probability of a farm having post-weaning diarrhoea.

**Variable**	**Estimate**^d^	**LCL^d ^95%**	**UCL^d ^95%**	***P***
Intercept	0.115	-0.992	1.222	0.84^a^
Feeding regimen: Feed restriction with two meals a day (F-R2M) vs. Three or more meals a day with or without feed restriction or *ad libitum *feeding (F-OTHER)	1.564	0.176	2.951	0.02^b^
Temperature control: Automatic vs. Manual	-1.306	-2.541	-0.072	0.03^b^
Number of sows^c^	0.0321	5.50 × 10^-4^	0.064	0.02^b^
A feeder as an only feed supply in a pen after weaning: Yes/No	1.277	-0.217	15.981	0.08^b^

LCL = Lower confidence limit UCL = Upper confidence limit

^a ^Significance estimated by t-test

^b ^Significance estimated by multiple Wald test

^c ^Number of sows is corrected to the average sow number in the data (n = 60).

^d ^Values are given in Log_e_-scale.

The presence of a feeder as an only feed supply in the weaning pen was included in the model because it improved the estimates of the other explanatory variables although it was not statistically significant (*P *= 0.08) in the multivariate analysis. Dispersion parameter for quasibinomial family was estimated to be 1.11. Null deviance of the model was 93.19 (df = 68) and residual deviance was 76.34 (df = 64).

Expected value of probability of experiencing moderate or severe problems related to post weaning diarrhoea can thus be described by:

$$\text{P}=\frac{{\text{e}}^{0.115+1.564\text{F}-1.306\text{A}+0.032(\text{NS}-60)+1.277\text{FPW}}}{{\text{1+e}}^{0.115+1.564\text{F}-1.306\text{A}+0.032(\text{NS}-60)+1.277\text{FPW}}}$$

where F is the feeding regimen (1 = two meals and feed restriction after weaning; 0 = other feeding regimens), A is the use of automatically regulated heating in the accommodation of weaners (1 = yes; 0 = no), NS is the number of sows on the farm and FPW is the use of a feeder as an only feed supply for newly-weaned pigs (1 = yes; 0 = no).

## Discussion

The feeding regimen after weaning, the presence of automatic temperature control in the accomodation for weaners and the number of sows on the farm had an effect on the occurrence of problems related to PWD.

The cases and the controls are considered representative based on the recognition of PWD, the herd size and the production level. Follow-up data was used to assign the case farms and the control farms related to the occurrence of post-weaning diarrhoea (PWD). PWD is a common enteric disorder of pigs and also well recognised by the farmers. In diagnostic samples sent to our laboratory from the farms participating in the follow-up, pathogenic *Escherichia coli *was a common finding in samples from piglets weaned during the previous fortnight [32]. The average herd size of the study farms was nearly the same as the average herd size on Finnish piglet producing farms taking part in a production monitoring system in 2000 [38]. Also according to the parameter of number of weaned pigs/sow/year the study farms were comparable to the farms taking part in the production-data recording system run by the Association of Rural Advisory Centres in 2000.

The risk of PWD was higher on the farms that fed the weaned piglets only twice a day with restricted amount of feed than on the farms that provided more than two meals per day with or without feed restriction or gave feed *ad libitum *after weaning. Our result related to the feeding regimen is in accordance with earlier results concerning the number of meals per day. With the amount of feed being similar, an increase in the number of meals per day has produced less severe signs of diarrhoea [25]. At this phase the weaned pigs can also be encouraged to eat more by increasing the number of meals per day or giving feed freely [39]. It is a common phenomenon in the immediate post-weaning period that pigs eat less than the optimum amount of feed [40] and it has been shown that at a farm level low feed intake after weaning is a risk factor for diarrhoea in weaners [24].

Despite the possible detrimental effects of low feed intake, restrictive feeding after weaning has been used as a preventive measure against PWD. In our study feed restriction as such in the immediate post-weaning period was not associated with PWD in the univariable analysis. In earlier studies overeating after weaning has been connected with the occurrence of PWD [16] and restriction of feed intake has reduced the incidence of and severity of post-weaning diarrhoea in early-weaned pigs [17,25,41,42] and in pigs weaned at the age of 5 weeks [43]. However, under farm circumstances restricted feeding can also predispose to diarrhoea. One explanation is that weaned pigs with a low feed intake may eat the excreta of their penmates and be exposed to a high number of faecal micro-organisms which then predisposes the pigs to infectious diarrhoea [31].

Automatic temperature control in the accommodation of weaners reduced the risk of PWD. Wide seasonal and diurnal variations in the temperature are typical for the climate in Finland [44] and therefore requirements on the temperature regulation capacity are high. A stable and optimal ambient temperature is very important during the two first weeks after weaning [45]. Temperature fluctuations have been associated with a greater incidence of post-weaning diarrhoea [46]. Skirrow [27] reported that large temperature fluctuations did not increase the risk of PWD, however, it is likely that ambient temperatures on Australian farms stay quite near to the optimum for weaned pigs despite an inadequate temperature control.

An increase in herd size was associated with a higher risk of post-weaning diarrhoea despite that our case and control farms were small compared with some other studies concerning herd size and enteric disease [26,47]. Our result is in contrast to a Danish study [26] in which risk of post-weaning diarrhoea decreased with increasing herd size. Production volumes and herd sizes vary between countries and according to the classification of the Danish study [26] most farms in our study would have been in the category of the smallest farms. In the Danish study it was thought that measures to decrease the risk of introducing infections when purchasing breeding stock and the management were better in bigger herds [26].

Factors such as hygiene [24,30], feed [15,30,48,49] and feed supplements [49-52] have been reported to affect the occurrence of PWD. In our study all farms except for two used commercial weaner feeds and therefore the type of feed could not be included in the analysis.

Due to the multifactorial etiology, finding case-specific preventive measures against PWD is a challenging task. Further studies are needed concerning different types of production systems to identify more predisposing factors and to define complex interactions between the factors relevant to PWD occurrence at a herd level. Our results should be regarded indicative for those factors that were found to increase the risk for PWD.

## Conclusion

Twice a day feeding of newly-weaned pigs should be avoided if the amount of feed given is restricted. After weaning the piglets should be fed at least three times a day or feed should be given *ad libitum*. Variation in ambient temperature should be minimized in housing of newly-weaned pigs and this can be achieved by using automatic temperature control. With increasing number of sows in the herds the risk of PWD increases and more attention should be paid to prevention of post-weaning diarrhoea.

## Abbreviations

AGPs: Antimicrobial growth promoters; PWD: Post-weaning diarrhoea

## Competing interests

The authors declare that they have no competing interests.

## Authors' contributions

TML has been involved in the initial design of the study and has been the main responsible for data analysis and drafting the manuscript, TL performed the statistical analysis and participated in the writing the manuscript, MY did most of the arrangements related to recruitment of the farmers and data acquisition and participated in the design of the questionnaires, MA was involved in the initial design and cooperation of the study and contributed to the writing of the manuscript. All authors have read and approved the final manuscript.
